# A Structure—Activity Relationship Study of the Inhibition of α-Amylase by Benzoic Acid and Its Derivatives

**DOI:** 10.3390/nu14091931

**Published:** 2022-05-05

**Authors:** Lei Guan, Haoyuan Long, Fazheng Ren, Yixuan Li, Hao Zhang

**Affiliations:** 1 Beijing Laboratory of Food Quality and Safety, College of Food Science and Nutritional Engineering, China Agricultural University, Beijing 100083, China; 2018306100420@cau.edu.cn (L.G.); fuxiao@cau.edu.cn (H.L.); 2 Department of Nutrition and Health, China Agricultural University, Beijing 100094, China; renfazheng@cau.edu.cn (F.R.); liyixuan@cau.edu.cn (Y.L.)

**Keywords:** phenolic acid, in vitro digestion, molecular docking, porcine pancreatic α-amylase, structure-activity relationship

## Abstract

Phenolic acids are widely found in fruits and vegetables. The inhibitory effect of phenolic acids on α-amylase, a key enzyme for starch digestion, has attracted the attention of researchers. To further investigate the effects of different substituents on the benzene ring of phenolic acid on the inhibition of α-amylase activity, in vitro experiments and molecular docking were used. The structure-activity relationships of 17 phenolic acids with benzoic acid as the parent nucleus were analyzed by determining their half inhibitory concentration (IC_50_) toward α-amylase. The results showed that 2,3,4-trihydroxybenzoic acid had the strongest inhibitory effect on α-amylase with an IC_50_ value of 17.30 ± 0.73 mM. According to the structure-activity analysis, the hydroxyl group at the 2-position on the benzene ring had a strong positive effect on the inhibitory activity of α-amylase, while methoxylation at the 2-position and hydroxylation at the 5-position had a negative effect. Molecular docking revealed that hydrogen bonding and hydrophobic interactions were involved in the inhibition, with hydrogen bonding being the primary force. These findings provide a more comprehensive understanding of phenolic acids as inhibitors of α-amylase and provide new ideas for the design of dietary formulations for diabetic patients.

## 1. Introduction

Diabetes mellitus (DM) is a chronic metabolic disease characterized by elevated blood glucose levels resulting from absolute or relative insulin deficiency within the body [1,2]. Persistent hyperglycemia can lead to a variety of complications that seriously affect human health and quality of life [3,4,5,6]. Type 2 diabetes mellitus (T2DM) is the most common type of diabetes mellitus, accounting for approximately 85% to 90% of all cases [7].

Pancreatic α-amylase is an important starch endo-hydrolase in the small intestine. It splits dextrinized starch into maltose and other oligosaccharides by catalyzing the hydrolysis of the α-1, 4-glucosidic bond. The products are further digested by other glucosidases [8]. Delaying the digestion of starchy foods by inhibiting α-amylase in the gastrointestinal tract, and thereby suppressing postprandial hyperglycemia, is an effective approach to reduce the impact of T2DM. Thus, the screening and identification of α-amylase inhibitors from natural products is of increasing interest to researchers hoping to advance the treatment and management of T2DM without the introduction of side effects.

Recent studies support the evidence that non-toxic and non-hazardous polyphenols, which are widely derived from plants, possess α-amylase inhibitory activity [9,10]. Additional work on the identification of natural α-amylase inhibitors should consider α-amylase-polyphenol interactions. Polyphenols are phenolic compounds that have one or more hydroxyl substitutions in one or two aromatic groups, and are divided into many subclasses, mainly phenolic acids and flavonoids. Natural plant phenolic acids primarily contain hydroxycinnamic acid, which has a C_6_-C_3_ structure, and hydroxybenzoic acid, which contains a C_6_ aromatic ring. By comparison, flavonoids consist of six aromatic rings. Free phenolic acids, such as benzoic acid, phenylacetic acid and cinnamic acid, have higher bioavailability and better water solubility than flavonoids [11]. 

The inhibitory effect of polyphenols on α-amylase is closely related to their molecular structure, where the substituents (hydroxyl and methoxy) of the aromatic rings contribute to their distinct molecular properties, such as polarity, stability and binding [12]. Therefore, we hypothesized that the structure of phenolic acid would likely influence its binding to α-amylase and directly affect its inhibitory activity and biological activity in vivo.

Phenolic acids with benzoic acid as the parent nucleus are the simplest structures in the phenolic acid family, and the in vitro inhibitory activity of some of these structures towards α-amylase has been determined in recent studies. For example, 2,5-dihydroxybenzoic acid (IC_50_ = 0.298 mM), gallic acid (IC_50_ = 1.25 mM) [13], vanillic acid (IC_50_ = 27.89 mM), 2,3,4-trihydroxybenzoic acid (IC_50_ = 33.33 mM), and syringic acid (IC_50_ = 44.81 mM) [14] have all been found to inhibit α-amylase. However, these inhibitory activities varied considerably depending on the type, number and position of the substituents on the benzene ring, and the intrinsic relationship between the structure and inhibition activity requires further investigation.

In this study, 17 pure phenolic acids with benzoic acid as the parent nucleus were selected for the analysis of the structure-activity relationship governing their binding, and to investigate their inhibition of α-amylase using in vitro inhibition activity experiments and molecular docking. These phenolic acids had the same benzoic acid core, but different types, numbers and arrangements of substituents on the aromatic ring. Half maximal inhibitory concentration (IC_50_) was used to characterize their inhibitory activity. Finally, the effects of the hydroxyl, methoxy and methyl groups of phenolic acid on the inhibition of α-amylase and their possible inhibition mechanisms will be discussed.

## 2. Materials and Methods

### 2.1. Materials

Porcine pancreatic α-amylase (EC 3.2.1.1; Catalog No. A3176) was purchased from Sigma-Aldrich Chemical Co. (St. Louis, MO, USA). Soluble starch (Catalog No. G83000) was purchased from Solarbio (Beijing, China). 3,5-dinitrosalicylic acid (DNS) reagent (Catalog No. PH1844) was purchased from Phygene Scientific (Fuzhou, China). Dimethyl sulfoxide (DMSO) was purchased from Guangfu Technology Development Co. (Tianjin, China). Benzoic acid and its derivatives were purchased from Xiya Reagent (Chengdu, China), Maklin Reagent (Shanghai, China), Aladdin (Shanghai, China), Shanghaiyuanye Bio-Technology Co. (Shanghai, China), Toronto Research Chemicals (Toronto, ON, Canada), HEOWNS (Tianjin, China) and Sigma-Aldrich Chemical Co. (St. Louis, MO, USA) Sodium chloride (NaCl), and potassium phosphate buffer was purchased from Yongda Chemical Reagent (Tianjin, China).

The properties of the phenolic acids and their derivatives, including the topological polar surface area (TPSA), the hydrogen bond acceptor/donor number, and the partition coefficient (XLogP3) were obtained from the PubChem public chemical database (https://pubchem.ncbi.nlm.nih.gov (accessed on 12 February 2022)).

### 2.2. Assessment of the In Vitro Inhibition of α-Amylase

The α-amylase inhibition assay was adapted from Phan et al. [15] with slight modifications. In order to simulate the small intestine conditions, a buffer pH of 6.8 and an incubation temperature of 37 °C were used. Porcine pancreatic α-amylase was dissolved in 0.2 M potassium phosphate buffer (pH 6.8). Phenolic acid solutions were prepared in 80% DMSO. The final concentration of DMSO in the reaction was 4%, which did not have an effect on the results and was consistent with our preliminary study. An aliquot of 50 μL of phenolic acid solution (10, 20, 30, 40 and 50 mM) was mixed with 400 μL of 0.2 M potassium phosphate buffer (pH 6.8 with 7 mM NaCl) in a 2 mL tube. After adding 100 μL of the enzyme solution (0.5 unit/mL), the tube was incubated at 37 °C for 5 min. Subsequently, 450 μL of the cooked soluble starch solution (5 mg/mL) was added to initiate the reaction at 37 °C for 20 min, followed by the addition of 500 μL of DNS reagent, and then the tubes were boiled for 15 min at 100 °C to inactivate the enzyme. A total of 200 μL of the reaction solution was transferred to a 96-well plate and the absorbance was measured at 540 nm using a microplate reader (Tecan Infinite 200 PRO; Tecan, Männedorf, Zürich, Switzerland). All of the samples were prepared and measured in triplicate. The inhibition rate was calculated using the following formula:

(1) $$\mathrm{Inhibition}\left(\%\right)=\left\{1-\frac{A\left[\mathrm{sample}\right]-A\left[\mathrm{blank}\right]}{A\left[\mathrm{control}+\right]-A\left[\mathrm{control}-\right]}\right\}\times 100$$

where:

A [control+] is absorbance at 100% enzyme activity (buffer with starch and enzyme);

A [control−] is starch background absorbance (buffer and starch without enzyme);

A [sample] is test sample absorbance (buffer with enzyme, starch and inhibitor);

A [blank] is inhibitor and starch background absorbance (a test sample without enzyme).

The inhibition rates were plotted against the logarithmic values of six concentrations of the test inhibitor and a non-linear regression analysis was employed to generate a dose-response curve and calculate the IC_50_ values (mM and mg/mL).

### 2.3. Molecular Docking

Molecular docking is a computational approach that provides information about the potential for binding between an inhibitor and an enzyme. The α-amylase crystal structure (PDB ID: 4W93) was downloaded from the RCSB PDB database (http://www.rcsb.org/pdb (accessed on 8 December 2021)). The three-dimensional structures of 17 phenolic acids were obtained from the PubChem chemical database. The ligand and water molecules were deleted from 4W93 to obtain a stable receptor for phenolic acid binding. The binding processes were visually analyzed using the CDOCKER algorithm in Discovery Studio 2019 software. In the CDOCKER docking process, the ligand was considered to be completely flexible and the protein was set as rigid [16,17,18]. Finally, the docking positions and the parameters (e.g., interaction energy and non-bond interaction distances) of the receptor and ligands were recorded. The binding energy was used in the results analysis. The protein target was considered to bind with the compound if the binding energy was less than −5 kJ∙mol^−1^ [19].

### 2.4. Statistical Analysis

All measurements were performed in triplicate and the results are presented as the mean ± standard deviation (SD). Differences were calculated by one-way ANOVA (analysis of variance) and post hoc LSD (least significant difference) tests using SPSS software (SPSS 26.0, Armonk, NY, USA). *p* values less than 0.05 were considered to be statistically significant.

## 3. Results and Discussion

### 3.1. In Vitro Inhibitory Activity of Phenolic Acids towards α-Amylase

The inhibitory activity of α-amylase increased with increasing phenolic acid concentration (Figure S1). The IC_50_ values are shown in Table 1 and were used to compare the inhibitory capacity of the analyzed compounds. 2,3,4-trihydroxybenzoic acid was the most potent inhibitor of α-amylase in this study (17.30 ± 0.73 mM), while 4-methylbenzoic acid was the weakest (52.35 ± 3.31 mM). The IC_50_ value of 4-hydroxy-3-methoxybenzoic acid was in agreement with that reported by Tan et al. [14]. The unfavorable acidity resulting from the presence of the phenolic acid in the solution may partially inhibit α-amylase activity, in addition to any active site-derived inhibition [20]. Moreover, when PBS was used as the buffer in a previous study [15], its buffering capacity was not sufficient to stabilize the pH of the reaction solution when the phenolic acid was tested at the highest concentration (Figure S2). Therefore, to avoid additional inhibition of the enzyme arising from the acidity of the phenolic acid in solution, a higher buffer concentration (0.2 M) than previously reported (0.02 M) was used to keep the pH variation in the reaction system within a reasonable range. This also resulted in the IC_50_ value of the phenolic acid being higher than that previously reported (3,4,5-trihydroxybenzoic acid, IC_50_ = 7.46 ± 0.24 mM; 3,4-dihydroxybenzoic acid, IC_50_ = 11.54 ± 0.45 mM; hydroxybenzoic acid, IC_50_ = 14.04 ± 0.65 mM) [13,14].

### 3.2. Effect of Hydroxylation on the Inhibition of α-Amylase

#### 3.2.1. The Number of Hydroxyl Groups

As shown in Table 1, the IC_50_ values of the phenolic acids with one or two hydroxyl groups on the benzene ring (4-hydroxybenzoic acid, 2,4-dihydroxybenzoic acid and 3,4-dihydroxybenzoic acid) were less than that of 3,4,5-trihydroxybenzoic acid, which has three hydroxyl groups (IC_50_ = 26.12 ± 1.89 mM). Besides, compared with 2,4-dihydroxybenzoic acid, the inhibition of 2,4,6-trihydroxybenzoic acid decreased when there was one more hydroxyl group. This is likely because the addition of hydroxyl groups reduces the hydrophobic properties of some phenolic acids, thus reducing their ability to pass through the entrance of the enzyme’s active site, which contains hydrophobic amino acid residues [21,22], making the inhibition of α-amylase less effective. However, the addition of hydroxyl groups enhanced the inhibitory activity of some compounds, such as 4-hydroxybenzoic acid, which had a significantly greater inhibitory capacity relative to benzoic acid. Additionally, 2,3,4-trihydroxybenzoic acid had an enhanced inhibitory capacity relative to 2,4-dihydroxybenzoic acid. For these structures, the hydroxyl groups may provide additional hydrogen binding opportunities to α-amylase that enhance their inhibitory activity [23]. It appears that simple changes to the number of hydroxyl groups have different effects on the ability of the phenolic acids to inhibit α-amylase. Therefore, the effect of the hydroxyl groups on the inhibitory ability of phenolic acid is likely to vary between the original structure and the hydroxylation position.

#### 3.2.2. The Position of the Hydroxyl Groups

**Figure 1:**
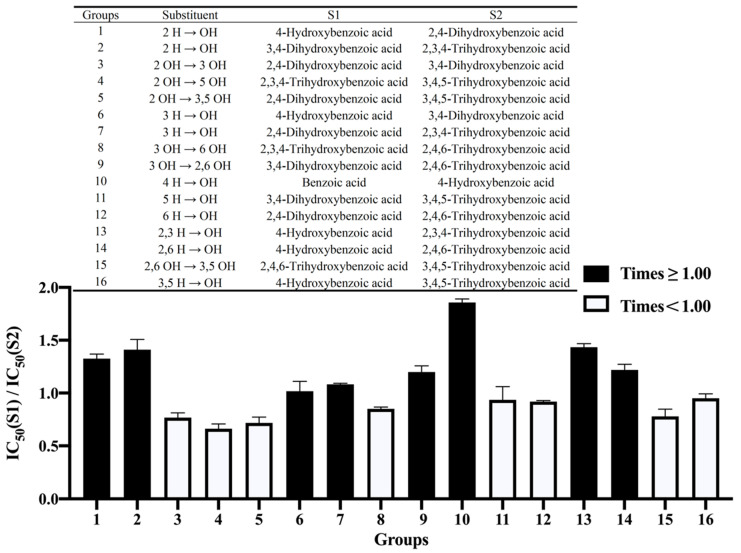
The effect of hydroxylation on the benzene ring on the inhibition of α-amylase by benzoic acid and its derivatives. S1—Structure 1; S2—Structure 2.

The inhibitory activity of benzoic acid and its derivatives against α-amylase seemed to depend on the hydroxylation position. Figure 1 shows the effects of the hydroxylation position on the inhibitive effect. The addition of a hydroxyl group at the 2-position resulted in a significant increase in the inhibition of α-amylase. For example, 2,4-dihydroxybenzoic acid inhibited α-amylase 33.31% more than 4-hydroxybenzoic acid (Group 1), and 2,3,4-dihydroxybenzoic acid inhibited α-amylase 46.56% more than 3,4-dihydroxybenzoic acid (Group 2). 

By comparison, hydroxylation at the 5-position of the benzene ring greatly weakened the inhibitory ability of the benzoic acid towards α-amylase. When the hydroxyl group at the 2-position of 2,3,4-trihydroxybenzoic acid was removed and a hydroxyl group was added at the 5-position, the inhibitory capacity decreased by 33.77% (Group 4). The addition of a hydroxyl group at the 5-position of 3,4-dihydroxybenzoic acid also resulted in a decrease in the inhibition of α-amylase (Group 11).

The above results indicated that the inhibition of α-amylase was positively affected by having a hydroxyl group at the 2-position of the benzene ring and negatively affected by the presence of a hydroxyl group at the 5-position. This may result from the formation of intramolecular hydrogen bonds with the carboxyl group through the hydrogen of the hydroxyl group and the carbonyl oxygen in the carboxyl group when the hydroxyl group is at the 2-position [24]. Intramolecular hydrogen bonding not only increases the stability of the phenolic acid itself, but also enhances its hydrophobicity [25]. Therefore, a hydroxyl group at the 2-position can enhance the ability of phenolic acid to infiltrate the hydrophobic binding pocket of α-amylase. Moreover, having an adjacent hydroxyl group has an important role in the inhibition of the enzyme [26], which may explain why 2,3,4-trihydroxybenzoic acid was the most potent inhibitor in our study. However, the 5-position hydroxyl group cannot form intramolecular hydrogen bonds with carboxyl groups [24], and the 5-position hydroxyl group may alter the electron cloud distribution of the phenolic acid [25]. These factors may negatively affect the inhibitory ability of phenolic acids towards α-amylase, but the exact mechanism remains to be investigated.

### 3.3. Effect of Methoxylation

**Figure 2:**
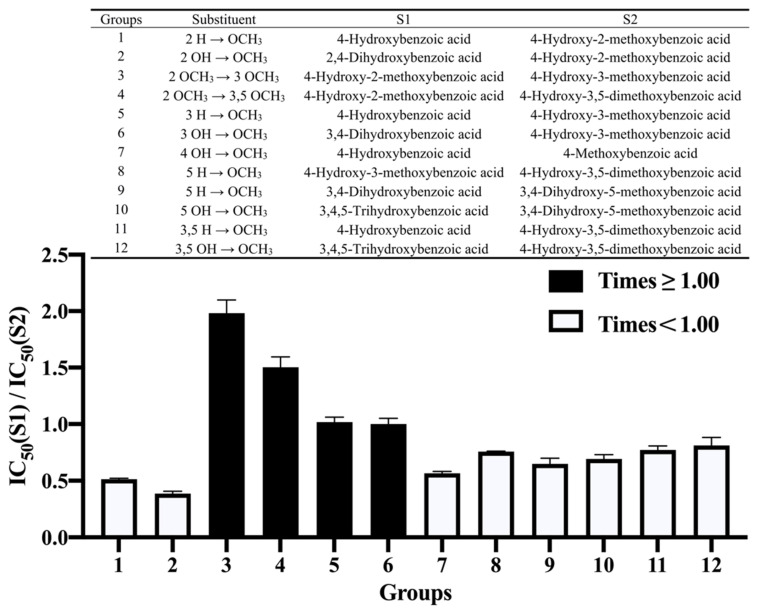
The effect of methoxylation on the benzene ring on the inhibition of α-amylase by benzoic acid and its derivatives. S1—Structure 1; S2—Structure 2.

The number of methoxy groups on the benzene ring did not appear to have a significant effect on the inhibition of α-amylase; thus, the discussion herein focuses on the position of methylation on the benzene ring. Figure 2 shows the effect of methoxylation on benzoic acid and its derivatives on the inhibition of α-amylase. The addition of methoxy groups at the 2-, 5- and 3,5-positions of the benzene ring resulted in a decrease in inhibitory activity (Groups 1, 8, 9 and 11). The effect of methoxylation at the 2-position was the largest, decreasing the inhibition by 47.46%. The methylation of the hydroxyl groups at the 2-, 4-, 5- and 3,5-positions had a negative effect on the inhibition (Groups 2, 7, 10 and 12). Among them, the effect at the 2-position was the strongest, and the reduction in inhibitory activity reached 60.59%. When the methoxy group at the 2-position was moved to the 3-position (Group 3), the inhibitory activity was enhanced to 200.76%. In summary, in all cases, methylation at the 2-, 4-, 5- and 3,5-positions resulted in decreased inhibitory activity of the phenolic acids, and the effect at the 2-position was the most prominent. Although the methylation of hydroxyl groups at some locations may reduce the polarity of the molecule, thereby enhancing the ability to enter the hydrophobic cavity of the enzyme, it also reduces the number of hydrogen-bonding donors and acceptors that play an important role in the inhibition [27,28,29].

### 3.4. Effect of Methylation

**Figure 3:**
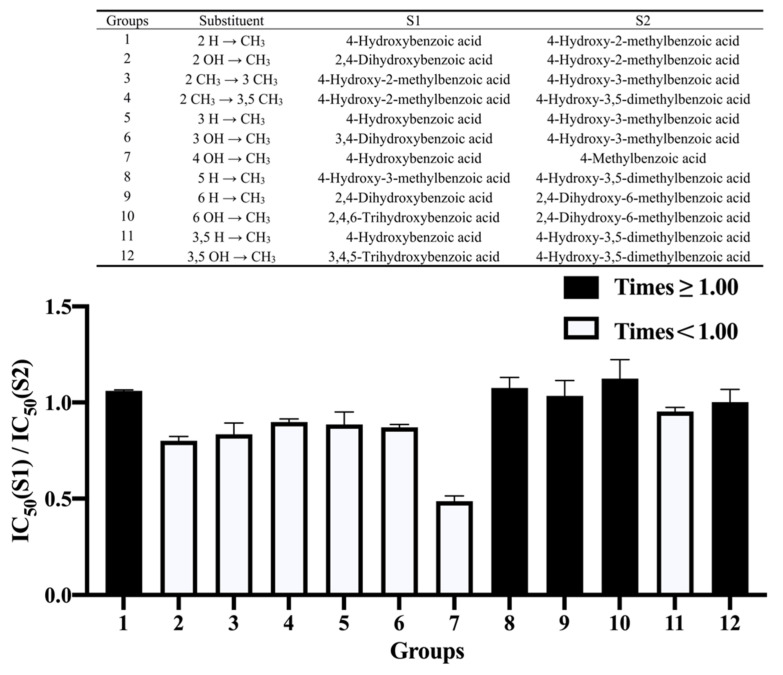
The effect of methylation on the benzene ring on the inhibition of α-amylase by benzoic acid and its derivatives. S1—Structure 1; S2—Structure 2.

According to Figure 3, in addition to the negative effect on the inhibition of replacing the hydroxyl at the 2- and 4-positions with a methyl group (Groups 2 and 7), the effect of the presence or absence of a methyl group at the other positions on the ring was not obvious. In addition, the benzene ring of 4-methylbenzoic acid had no hydroxyl group, but had a methyl group with negative influence, making it the weakest inhibitor in our study. In previous reports, both the substitution of hydroxyl groups with methyl groups and the addition of a methyl group at certain positions reduced the inhibitory activity of flavonoids against α-amylase [12], because replacing a hydroxyl group with a methyl group will reduce the formation of a hydrogen bond, which plays an important role in inhibition. In our study, this effect did not seem to be applicable to these phenolic acids with benzoic acid as the parent nucleus.

### 3.5. Relationship between the Properties of the Phenolic Acids and Their IC_50_ Values

#### 3.5.1. Topological Polar Surface Area

**Figure 4:**
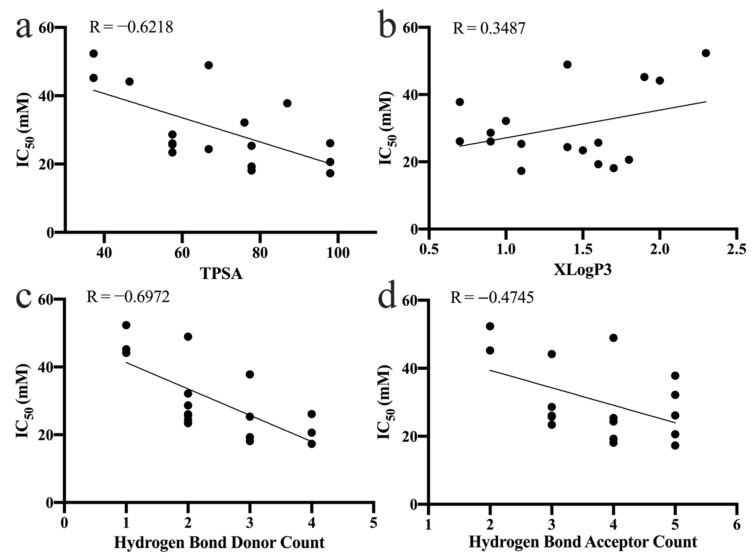
Relationship between the α-amylase IC_50_ values and (**a**) the TPSA, (**b**) the partition coefficient (XLogP3), (**c**) the hydrogen bond donor number, and (**d**) the hydrogen bond acceptor number of the phenolic acids. R—Pearson correlation coefficient.

The topological polar surface area (TPSA) represents the total surface of polar atoms in a molecule, and is a useful indicator to describe the bioaccessibility and bioavailability of a compound in vivo [30,31,32]. Notably, the inhibition of α-amylase by phenolic acids occurs mainly in the small intestine, so it is important to consider both the bioavailability and inhibition of a compound when assessing its potency [33]. In the present study, we found a negative correlation between the TPSA and the inhibitory activity (IC_50_ values) of the phenolic acids against α-amylase (Figure 4a). This suggests that phenolic acids with higher inhibitory activity also have higher bioavailability, and thus inhibit α-amylase more effectively.

#### 3.5.2. Partition Coefficient

According to the PubChem chemistry database, the hydrophobicity of the phenolic acids was assessed using their partition coefficient (XLogP3). The XLogP3 values of the phenolic acids were positively correlated with their IC_50_ values (Figure 4b). The linear regression equation was IC_50_ = 8.260 XLogP3 + 18.87 (R = 0.3487). As shown by the equation, hydrophobic forces are one of the ways by which phenolic acids bind to α-amylase. The inhibition of α-amylase decreased with an increase in the partition coefficient. Therefore, the binding of phenolic acid to α-amylase was not primarily because of hydrophobic forces, which is consistent with what has been reported for flavonoids in previous studies [9]. Our results also suggest that the methylation of hydroxyl groups at the 2-, 4-, 5- and 3,5-positions on the benzene ring of the phenolic acids weakened their ability to inhibit α-amylase.

#### 3.5.3. Number of Hydrogen Bond Donors and Acceptors

To investigate the role played by hydrogen bonding in the binding of phenolic acids to α-amylase, the relationship between the number of hydrogen bond donors/acceptors of the phenolic acid and the IC_50_ values for the inhibition of α-amylase was analyzed (Figure 4c,d). There was a negative correlation between the IC_50_ value for α-amylase and the number of hydrogen bond donors/acceptors of the phenolic acid. That is, the inhibitory activity of the phenolic acid increased with an increase in the number of hydrogen bond donors/acceptors. This result suggests that hydrogen bonding is very important for the binding of phenolic acid to α-amylase.

### 3.6. Molecular Docking

**Figure 5:**
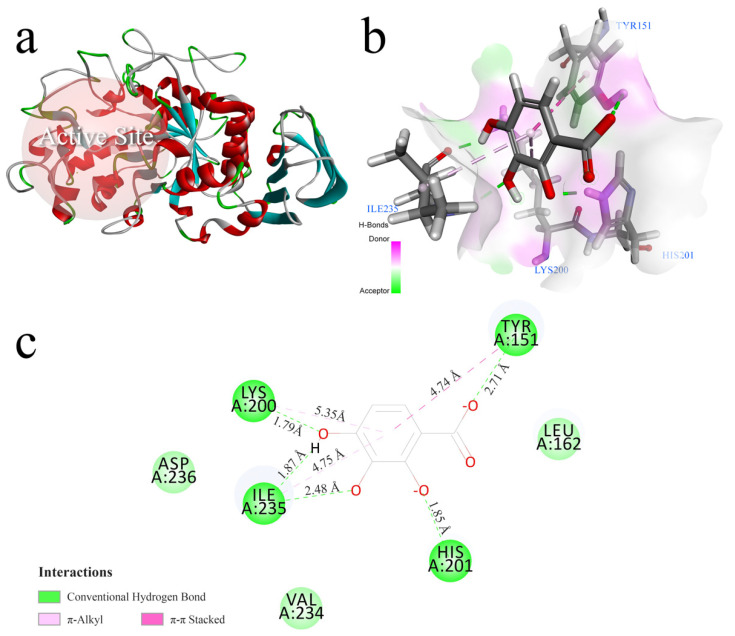
(**a**) The α-amylase crystal structure (4W93) and docking site (also the active site). The molecular docking results of 2,3,4-trihydroxybenzoic acid with α-amylase: (**b**) the best docking pose of 2,3,4-trihydroxybenzoic acid in 4W93, and (**c**) the binding mode of 2,3,4-trihydroxybenzoic acid with 4W93.

To further understand the binding mode, binding site, and binding energy between the phenolic acids and α-amylase, molecular docking was performed. The α-amylase docking site for phenolic acid, as predicted by the docking, is shown in Figure 5a. Glu 233, Asp 197, and Asp 300 are conserved amino acids found in the catalytic pocket of α-amylase [34], and these amino acid residues are also included in the docking site. Therefore, the region where the phenolic acids docked with α-amylase can also be considered to be an active site of α-amylase. The binding of phenolic acids to α-amylase is often considered to be an exothermic process [35], and thus the binding free energy (E_b_) of phenolic acid α-amylase docking is usually a negative value. In line with the CDOCKER protocol, the E_b_ value of docking is shown as the CDOCKER_Interaction_Energy in Table S1.

Combined with the results of the in vitro experiments, 2,3,4-trihydroxybenzoic acid, with a high CDOCKER score (CDOCKER_Interaction_Energy = −27.9624 kJ∙mol^−1^) corresponding to both the most stable conformation and the lowest IC_50_ value, was selected for the analysis of the interactions between α-amylase and the phenolic acids. According to the three-dimensional docking pattern (Figure 5b) and the two-dimensional schematic (Figure 5c), it was clear that 2,3,4-trihydroxybenzoic acid was inserted into the cavity of the active site of α-amylase. The amino acid residues interacting with 2,3,4-trihydroxybenzoic acid included Lys 200, Tyr 151, Ile 235 and His 201. Two π-alkyl interactions and one π–π stacking interaction were found between 2,3,4-trihydroxybenzoic acid and the amino acid residues Lys 200, Ile 235 and Tyr 151. Additionally, these hydrophobic interactions between the benzene ring of 2,3,4-trihydroxybenzoic acid and the amino acid residues formed a Y-shaped orientation that should strengthen the backbone of the phenolic acid-α-amylase complex. In this way, the inhibitory activity was enhanced. 2,3,4-trihydroxybenzoic acid formed five hydrogen bonds with the amino acid residues Lys 200, Tyr 151, Ile 235, and His 201. Figure 5c shows the distances of the three hydrophobic forces (4.74 Å, 4.75 Å and 5.35 Å) and the five hydrogen bonds (1.79 Å, 1.85 Å, 1.87 Å, 2.48 Å and 2.71 Å). The predominant force was hydrogen bonding, which is consistent with the fact that 2,3,4-trihydroxybenzoic acid was the most potent inhibitor. In particular, His 201 interacted with 2,3,4-trihydroxybenzoic acid via hydrogen bonding, and contributed to the inhibition of α-amylase. Overall, phenolic acids with benzoic acid as the parent nucleus are hypothesized to bind to α-amylase primarily through hydrogen bonding, which is consistent with other studies on the interaction of α-amylase with phenolic compounds [36,37].

## 4. Conclusions

In this research, the structure-activity relationship between phenolic acids with benzoic acid as the parent nucleus and α-amylase was investigated by using in vitro inhibition assays and molecular docking. The results showed that the hydroxyl group at the 2-position of the benzene ring had a positive effect on the inhibitory activity, while the introduction of a methoxy group at the 2-position of the benzene ring and a hydroxyl group at the 5-position had a negative effect. The introduction of a methyl group on the benzene ring had no obvious effect on the inhibition. The predominant interaction between α-amylase and the phenolic acids was hydrogen bonding, whereas hydrophobic forces did not seem to play a significant role in the interactions. Molecular docking provided valuable details about the binding interactions of benzoic acid and its derivatives with α-amylase. This study provides new insights into the mechanism of α-amylase inhibition by phenolic acids.

## Figures and Tables

**Table 1:** The structures of benzoic acid and its derivatives and their IC50 values for α-amylase.

Basic Structure	Compound No.	Substitution			IC_50_ Values against α-Amylase	
		OH	OCH_3_	CH_3_	mM	mg/mL
** 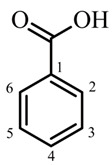 **	1				45.25 ± 2.15	5.525 ± 0.263
	2	4			25.72 ± 1.33	3.552 ± 0.184
	3	2,4			19.29 ± 1.18	2.973 ± 0.182
	4	3,4			25.36 ± 1.23	3.908 ± 0.190
	5	2,3,4			17.30 ± 0.73	2.943 ± 0.124
	6	2,4,6			20.62 ± 0.53	3.508 ± 0.090
	7	3,4,5			26.12 ± 1.89	4.444 ± 0.332
	8			4	52.35 ± 3.31	7.127 ± 0.450
	9	4		2	23.40 ± 1.11	3.560 ± 0.169
	10	4		3	28.65 ± 0.76	4.359 ± 0.116
	11	4		3,5	26.05 ± 1.00	4.328 ± 0.167
	12	2,4		6	18.12 ± 1.30	3.047 ± 0.219
	13		4		44.15 ± 1.39	6.717 ± 0.212
	14	4	2		48.95 ± 2.51	8.230 ± 0.422
	15	4	3		24.38 ± 0.71	4.099 ± 0.119
	16	3,4	5		37.82 ± 1.59	6.965 ± 0.292
	17	4	3,5		32.16 ± 0.88	6.372 ± 0.175

## Supplementary Materials

The following supporting information can be downloaded at https://www.mdpi.com/article/10.3390/nu14091931/s1, Figure S1: Inhibitory effect of 2,3,4-trihydroxybenzoic acid on the activity of α-amylase; Figure S2: The effect of the maximum concentration of benzoic acid and its derivatives on the pH value of the reaction system before adjusting the concentration of the PBS buffer; Table S1: CDOCKER Energy and CDOCKER Interaction Energy data for benzoic acid and its derivatives.
