# Supplementary material for: A Structure—Activity Relationship Study of the Inhibition of α-Amylase by Benzoic Acid and Its Derivatives
Source: Nutrients. 2022 May 5;14(9):1931. doi: 10.3390/nu14091931 (PMC9102017; doi:10.3390/nu14091931)
Supplement: Supplementary file 1 [file nutrients-14-01931-s001.zip › nutrients-1680283-supplementary.pdf]

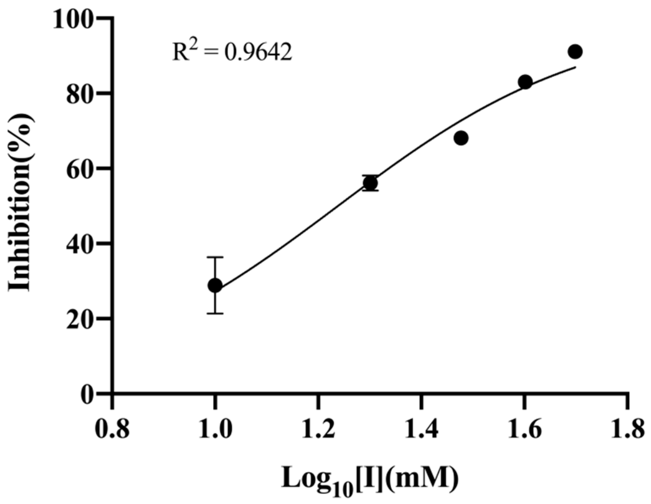

Figure S1. Inhibitory effect of 2,3,4-trihydroxybenzoic acid on the activity of α-amylase.

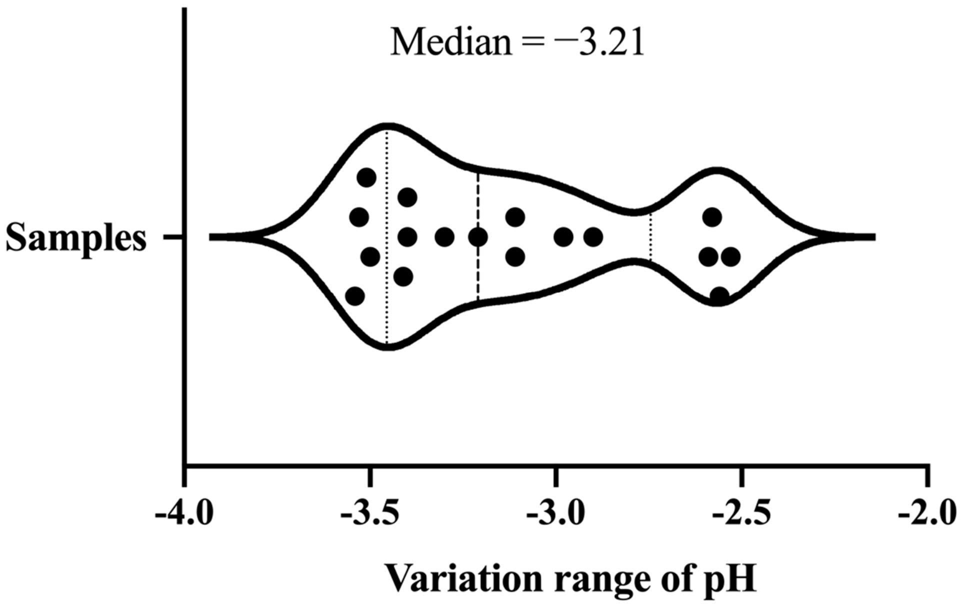

Figure S2. The effect of the maximum concentration of benzoic acid and its derivatives on the pH value of the reaction system before adjusting the concentration of the PBS buffer.

Table S1. CDOCKER Energy and CDOCKER Interaction Energy data for benzoic acid and its derivatives.

Compound No.	Compound Name	CDOCKER Energy * (kcal·mol^{-1})	CDOCKER Interaction Energy * (kcal·mol^{-1})
1	Benzoic acid	−13.0580	−15.5730
2	4-Hydroxybenzoic acid	−18.3349	−19.0955
3	2,4-Dihydroxybenzoic acid	−16.5076	−17.9185
4	3,4-Dihydroxybenzoic acid	−23.7652	−19.9858
5	2,3,4-Trihydroxybenzoic acid	−17.7669	−27.9624
6	2,4,6-Trihydroxybenzoic acid	−16.5577	−18.5385
7	3,4,5-Trihydroxybenzoic acid	−22.1409	−20.6498
8	4-Methylbenzoic acid	−16.1707	−18.1230
9	4-Hydroxy-2-methylbenzoic acid	−20.5714	−25.4675
10	4-Hydroxy-3-methylbenzoic acid	−19.0811	−20.4046
11	4-Hydroxy-3,5-dimethylbenzoic acid	−21.6985	−23.6935
12	2,4-Dihydroxy-6-methylbenzoic acid	−14.9262	−18.8302
13	4-Methoxybenzoic acid	−15.0808	−19.6385
14	4-Hydroxy-2-methoxybenzoic acid	−16.3666	−24.9607
15	4-Hydroxy-3-methoxybenzoic acid	−19.6819	−21.8122
16	3,4-Dihydroxy-5-methoxybenzoic acid	−24.6296	−27.9799
17	4-Hydroxy-3,5-dimethoxybenzoic acid	−10.1005	−25.4468

* The CDOCKER Energy and CDOCKER Interaction Energy for the top ranked binding poses of 17 phenolic acids obtained after conducting the molecular docking studies on the α-amylase (PDB ID: 4W93 using the CDOCKER algorithm in the software Discovery Studio 2019, BIOVIA Inc., San Diego, CA, USA).
